# Molecular Insights Into Regulatory T-Cell Adaptation to Self, Environment, and Host Tissues: Plasticity or Loss of Function in Autoimmune Disease

**DOI:** 10.3389/fimmu.2020.01269

**Published:** 2020-09-15

**Authors:** Cheryl Y. Brown, Timothy Sadlon, Christopher M. Hope, Ying Y. Wong, Soon Wong, Ning Liu, Holly Withers, Katherine Brown, Veronika Bandara, Batjargal Gundsambuu, Stephen Pederson, James Breen, Sarah Anne Robertson, Alistair Forrest, Marc Beyer, Simon Charles Barry

**Affiliations:** ^1^Molecular Immunology, Robinson Research Institute, University of Adelaide, Adelaide, SA, Australia; ^2^Women's and Children's Health Network, North Adelaide, SA, Australia; ^3^Bioinformatics Hub, University of Adelaide, Adelaide, SA, Australia; ^4^QEII Medical Centre and Centre for Medical Research, Harry Perkins Institute of Medical Research, Murdoch, WA, Australia; ^5^German Center for Neurodegenerative Diseases (DZNE), Bonn, Germany

**Keywords:** Treg FOXP3, gene regulation, genetic risk of disease, T-cell fate, T-cell plasticity

## Abstract

There has been much interest in the ability of regulatory T cells (Treg) to switch function *in vivo*, either as a result of genetic risk of disease or in response to environmental and metabolic cues. The relationship between levels of FOXP3 and functional fitness plays a significant part in this plasticity. There is an emerging role for Treg in tissue repair that may be less dependent on FOXP3, and the molecular mechanisms underpinning this are not fully understood. As a result of detailed, high-resolution functional genomics, the gene regulatory networks and key functional mediators of Treg phenotype downstream of FOXP3 have been mapped, enabling a mechanistic insight into Treg function. This transcription factor-driven programming of T-cell function to generate Treg requires the switching on and off of key genes that form part of the Treg gene regulatory network and raises the possibility that this is reversible. It is plausible that subtle shifts in expression levels of specific genes, including transcription factors and non-coding RNAs, change the regulation of the Treg gene network. The subtle skewing of gene expression initiates changes in function, with the potential to promote chronic disease and/or to license appropriate inflammatory responses. In the case of autoimmunity, there is an underlying genetic risk, and the interplay of genetic and environmental cues is complex and impacts gene regulation networks frequently involving promoters and enhancers, the regulatory elements that control gene expression levels and responsiveness. These promoter–enhancer interactions can operate over long distances and are highly cell type specific. In autoimmunity, the genetic risk can result in changes in these enhancer/promoter interactions, and this mainly impacts genes which are expressed in T cells and hence impacts Treg/conventional T-cell (Tconv) function. Genetic risk may cause the subtle alterations to the responsiveness of gene regulatory networks which are controlled by or control FOXP3 and its target genes, and the application of assays of the 3D organization of chromatin, enabling the connection of non-coding regulatory regions to the genes they control, is revealing the direct impact of environmental/metabolic/genetic risk on T-cell function and is providing mechanistic insight into susceptibility to inflammatory and autoimmune conditions.

## Introduction

### Establishing and Maintaining Immune Homeostasis

To maintain health, the immune system continuously and dynamically balances robust reactivity against pathogens with tolerance or unresponsiveness to self-antigens, commensal bacteria, food, and external harmless antigens (1). This is in part mediated by the effector arm of the adaptive immune system, and two of the major T-cell mediators of this are CD8 and CD4 cells. These are selected based on their ability to respond to antigens presented on either MHC class 1 or 2, respectively. The CD4+ T-cell compartment comprises a growing number of specific effector subsets, each of which is programmed to respond to defined antigen families and home to specific locations. Antigen specificity is determined by the affinity and avidity of T-cell receptors (TCRs). This specificity is generated during CD4/CD8 commitment, differentiation, and selection in the thymus, in a process that includes deletion of (strongly) self-reactive TCR-bearing T cells as a mechanism to prevent autoreactive TCR-bearing clones being released into the periphery. As no biological process is 100% efficient, there is the potential for self-reactive T cells to escape selection and hence be released into the periphery. To manage this, regulatory T cells (Treg) are also generated with the same TCR specificities. Treg are selected in the thymus but also generated from naïve T cells in the periphery. Both thymic (nTreg) and induced (pTreg) Treg are similar in function, but they have different roles and targets cells (2, 3). The key difference between these subsets is that pTreg provide immune surveillance of specific organs and biological processes in the periphery for which there is no inherited specificity, such as tolerization of the conceptus in pregnancy or the bacteria and food antigens in the gut by pTreg (3).

### Roles and Function of Treg

In a general sense, Treg act as “policemen” of the immune system to limit rogue immune activity, and this role in immune homeostasis is critical. In addition to regulating antigen-specific immune responses, Treg are capable of regulating cell function in an antigen-independent manner (4) and are now implicated in tissue homeostasis and repair (5–8). Treg actively control the proliferation and activation of cells of both the adaptive and innate immune systems and achieve this using multiple mechanisms, which are tailored to the environment in which they are required to function (1). The suppressor mechanism is likely to differ according to the physiological and inflammatory state encountered (1, 9). While autoimmunity and chronic inflammation are accepted to arise as a general failure of tolerance, given that the effector and Treg arms of the system need to be in balance, this can occur because of numerical reduction in Treg, functional reduction in Treg potency without reduced numbers, expansion of effector T cells, or T effector resistance to suppression. In order to examine this at high resolution in clinical cohorts, Treg-specific biomarkers are essential. The first biomarker for Treg was CD25, the IL2 receptor alpha chain, which gained widespread recognition as stable expression of the IL2 receptor (CD25) tracks with the suppressor function in CD4+ T cells (10). Many groups have further characterized CD25 expression and conclude that CD25 expression is strongly upregulated on Treg, but transient activation of CD4+ T cells can induce CD25 without inducing regulatory function, so it is not an exclusive Treg functional marker. The definitive biomarker of suppressor function is FOXP3, but because it requires an intracellular stain with a protocol to fix and permeabilize cells, viable cells cannot easily be recovered, making it less tractable as a live cell biomarker. In search of surface-expressed surrogates of the suppressor function or FOXP3 expression, two groups observed that reduced expression of the IL7 receptor (CD127) is a hallmark of the human Treg phenotype (11, 12). However, as activated murine Treg express CD127 strongly (13), CD127 is not selective for mouse Treg. Deeper interrogation of the function of Treg subsets is suggesting that differential expression of other cytokine/chemokine receptors on Treg may be useful for tracking Treg *ex vivo*. A growing number of other cell surface markers are found on specific Treg subsets, e.g., TIGIT (14–16), FcRL3 (17), GARP/LRRC32 (18–21), CD73 and CD39 (22, 23), and, more recently, PI16 (24). The mechanism for TIGIT in establishing the suppressor function both directly and indirectly includes induction of tolerogenic dendritic cells (14, 15), and coexpression with FcRL3 marks human memory Treg that express Helios and are highly suppressive (17). Many of these genes are regulated by FOXP3.

Because many of these cell surface molecules are also found on effector cell populations, they are not powerful biomarkers in isolation, and more complexity in the Treg phenotype exists than two parameter biomarker combinations suggest. The use of new single-cell transcriptomic approaches (25) and high-resolution cytometry (26, 27) is enabling better resolution of the coexpression of marker genes in these low-abundance Treg subsets.

### Molecular Mechanisms Shaping Treg Stability and Phenotype

FOXP3 is the key transcription factor for the formation and function of Treg in mice and humans (28–30). Genomics including RNAseq and chromatin immunoprecipitation (ChIP) has helped identify the molecular basis for the action of FOXP3 in shaping regulatory T cells and in establishing and maintaining lifelong tolerance. While loss of Treg function is observed in a wide variety of autoimmune and chronic inflammation states, there remains the possibility that the loss of function is a consequence of, and not the cause of, autoimmunity and chronic inflammation. It is clear that Treg lineage formation is dependent on FOXP3, which establishes and maintains suppressor function. With additional analysis of FOXP3 cell origins in mice, the existence of two FOXP3+ve Treg populations of different ontologies has revealed the mechanism for tolerance induction in the periphery. Natural or thymic Treg emerge from the thymus stably expressing FOXP3 and fully mature. In contrast, peripheral Treg arise from FOXP3-negative naïve T cells which do not express FOXP3 until stimulated in the presence of cytokines and transcriptional activators, which turn on the FOXP3 gene. The molecular steps required to set up and stabilize the expression of FOXP3 in the thymus, including a key role played by SATB1 (31–33) and Helios (34–39), may be distinct from those inducing FOXP3 in the periphery. Recently, the role of Helios in Treg ontology was further elucidated as deficiency in Helios results in preferential differentiation into pTreg (35). Once established, many of the gene regulatory networks (GRNs) controlled by FOXP3 are similar in pTreg and tTreg, and subtle functional gene networks are set up to shape lineage restriction or maturation state. Hence, using similar molecular mechanisms, the Treg compartment has the ability to acquire tolerance to antigens from inherited repertoires, such as self-tissues and organs (tTreg), and to *de novo* antigen exposure, such as pregnancy alloantigens, commensal bacteria, food, and chemicals (pTreg).

### Control of Expression of FOXP3

During formation of Treg in the thymus, the FOXP3 locus is set up for active transcription by chromatin remodeling (31, 32), and the protein SATB1 is implicated in initiating this. Other transcription factors, including FOXP1 (40), are also required to set the stable expression of FOXP3. The expression of FOXP3 is impacted at the transcription and posttranscriptional levels, and this is sensitive to reversible processes including methylation (41–48) and acetylation (49–51). Additional regulation of FOXP3 gene expression is influenced by non-coding RNA-mediated mechanisms (33, 52–59). The regulation of transcription of FOXP3 by distinct modules including the Treg-specific demethylated region (TSDR) (41, 42, 60) has revealed marks for FOXP3 expression control and can discriminate between thymic Treg FOXP3 expression and activation-dependent expression of FOXP3 in naïve T cells in the periphery (41, 47). The methylation or demethylation of the TSDR is controlled by DMT3 or TET, respectively, and this process is regulated tightly in both thymic induction of FOXP3 and induction of FOXP3 in the periphery (61–63). Detailed functional mapping of the FOXP3 locus regulatory elements has defined specific regions near the TSDR identified as conserved non-coding sequences (CNS) 1, 2, and 3 (64). CNS1 restricts expression of FOXP3 to iTreg. CNS2 includes the TSDR and drives maintenance of FOXP3 in all Treg, and CNS3 is responsible for FOXP3 expression in thymic Treg (64). Specific transcription factors bind at each region, including AP1 and NFAT at CNS1 (60), Runx1 and CBFβ at CNS2 (65), and cRel at CNS3 (64). Activation-induced expression of FOXP3 in naïve human CD4+ T cells (66–68) results from partial but not complete demethylation of the FOXP3 locus, generating iTreg (41). In the presence of TGFβ and all-trans retinoic acid (ATRA) (64), the expression of FOXP3 is stabilized to some degree. Hence, the relative methylation state of the FOXP3 regulatory elements (CNS1, CNS2, and CNS3) is a potential axis for Treg plasticity.

### Molecular Identification of the FOXP3 Regulome

**Figure 1:**
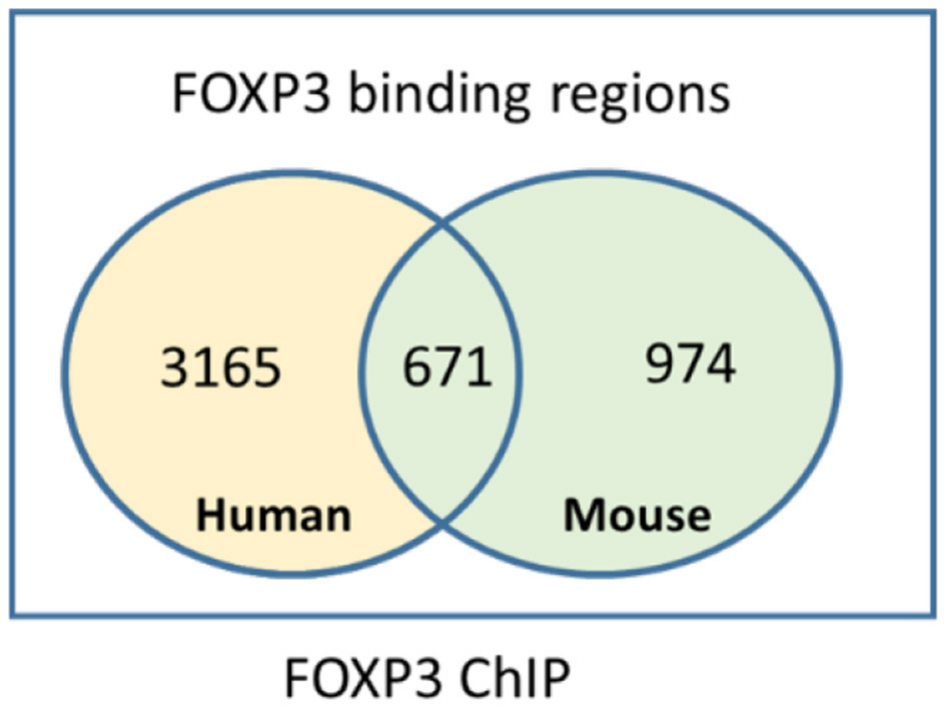
Intersection of mouse and human FOXP3 target genes identified by chromatin immunoprecipitation.

Understanding the mechanisms of transcriptional control of the Treg suppressor genotype by FOXP3 has been increased by ChIP experiments, which crosslink transcription factors bound to genomic DNA. Genome-wide mapping of FOXP3-binding sites provides insight into the regulation of the genes that shape the Treg phenotype. In human Treg, of the 2,000–3,000 regions bound by FOXP3 identified by our and other FOXP3 ChIP experiments (57, 58, 69, 70), only a subset of the FOXP3-bound regions maps to genes that are directly differentially expressed or repressed in human Treg at any given time, including SATB1 (33). FOXP3 ChIP studies have identified a significant number of loci in mouse and human Treg that are directly bound by FOXP3 and can be annotated to differentially expressed Treg genes (Figure 1). However, many loci either were too far from a transcription start site to annotate to a target gene easily or do not appear to be associated with differentially expressed genes in Treg. This can be explained because there are a number of differentially expressed genes in Treg that are indirect targets of FOXP3 or are controlled by FOXP3-induced miRNAs. For example, in our human FOXP3 ChIP dataset, only 750 of almost 3,000 FOXP3-bound regions were annotated to a differentially expressed gene in human Treg (57). This revealed a network of core genes that are tightly regulated by FOXP3. However, there is a limitation of linear models of nearest-neighbor annotation, as it does not capture interactions that occur as a result of DNA looping. Nonetheless, specific genes interact with FOXP3 to form the FOXP3 GRN, and this GRN shapes the function of Treg.

### Multiplexed Transcriptional Control of T-Cell Function

**Figure 2:**
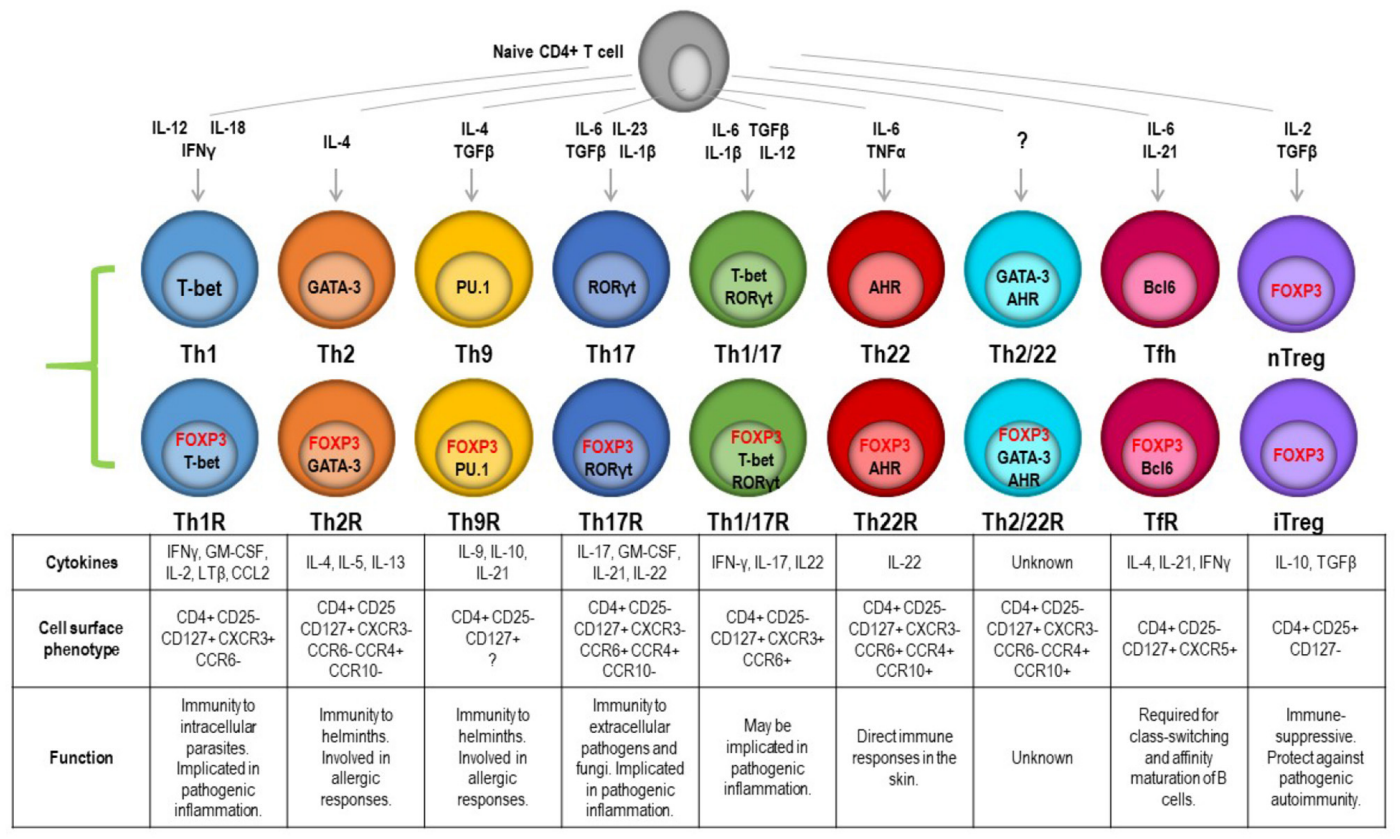
Transcription factor-controlled function in both effector and Treg lineages gives rise to paired Treg to match effectors. These can be resolved by combinatorial chemokine receptor profiling and are predicted to be able to follow the same cues into specific tissues to manage a pathogen and the restoration of homeostasis once the pathogen is eliminated.

Each helper lineage in the CD4 pool has a defining transcription factor, the expression of which shapes lineage-restricted function. As previously stated, FOXP3 controls the GRN essential for suppressor function, but this also acts in the context of the lineage-defining transcription factors. There can hence be a second or third partner transcription factor working in cooperation with the lineage-defining master regulator. These transcription factors can be induced by specific external stimuli, including cytokines and growth factors, metabolites, and cell contact-mediated signals. As a result, a transcriptional program is established which enables the cell to express pathogen-specific effector molecules and follow pathogen-specific homing cues. This raises the possibility that terminal differentiation and function may not be predetermined or fixed in a given lineage but is reprogrammable. A requirement for plasticity in T-cell responses may be 2-fold; it may be part of a mechanism to quell an active immune response once the pathogen has been cleared. Alternately, functional plasticity may enable multiple tailored responses from a common progenitor, giving the option of a response tuned to the challenge type and site. An emerging theme is that the Treg compartment is paired with the conventional T-cell (Tconv) effector compartment so a matching Treg can regulate any immune response mediated by any T-cell subset (24, 71–73). This is supported by the detection of lineage-specific transcription factors [e.g., Tbet (74) and IRF4 (75)] coexpressed with FOXP3 in Treg subsets, and this has been validated in various mouse models (Figure 2). The application of single-cell RNAseq, CITEseq, and other high-resolution transcriptomics on highly purified human Treg subsets is enabling the identification of more signature molecules for each functional subset. Single-cell transcriptomics has recently been applied to Treg from lymphoid and non-lymphoid tissues to compare differences and similarities in rare tissue-homing populations, and this reveals common non-lymphoid tissue-specific signatures in Treg but also that there are significant differences between these subsets in mice and humans (25). The use of functional assays to interrogate highly purified cell populations then allows the question of altered committed lineage proportions vs. plasticity to be better understood.

### Non-coding RNAs: Rheostats of Treg Gene Expression

**Figure 3:**
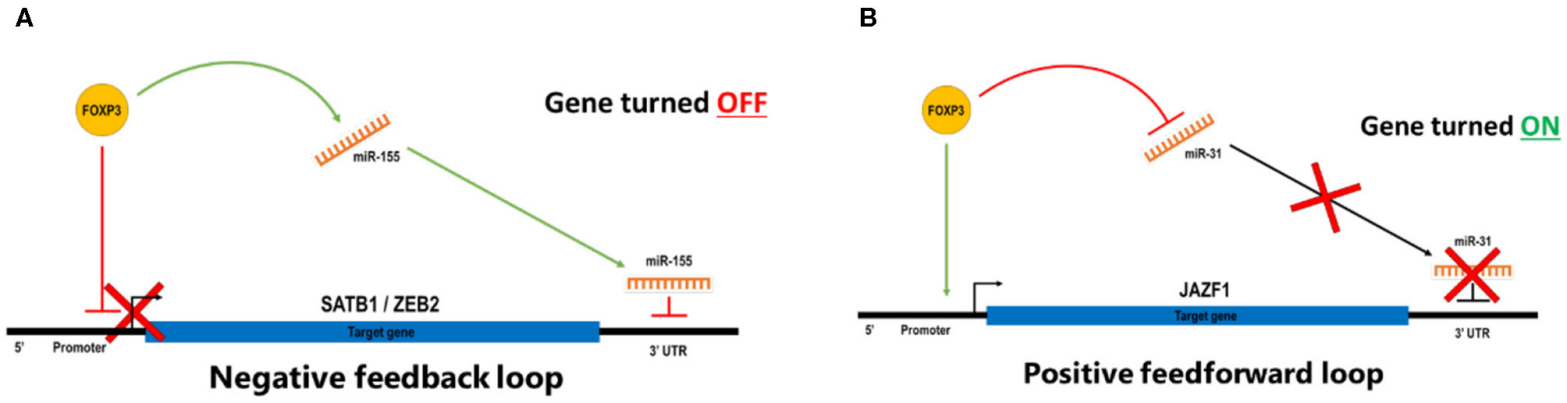
**(A)** In a negative feedback loop, FOXP3 binds directory to the target genes to repress transcription and also induces miRNA that targets the 3′ UTR of the same genes to degrade transcripts or blockade of translations. **(B)** In a positive feedforward loop, FOXP3 binds directory to the target genes to induce transcription and also represses miRNA that targets the 3′ UTR of the same genes to prevent degradation of transcripts or blockade of translations. In a stable Treg, FOXP3 represses miR-31 by direct binding to regulatory elements associated with the gene, and FOXP3 also targets the promoter of a suppressor function reinforcing the gene to turn it on. In effector T cells, miR-31 expression prevents the expression of FOXP3 by targeting FOXP3 mRNA for degradation.

MicroRNAs (miRNAs) are 21–22-nt, non-coding RNAs often found within the introns of genes. They can posttranscriptionally regulate gene expression by either cleavage of the mRNA transcript or inhibition of translation. The impact of a small number of regulated miRNAs is significant because a single miRNA can target and regulate multiple genes. Likewise, multiple miRNAs can target an individual mRNA transcript, giving rise to complex regulatory networks and fine-tuning of gene dose. Rather like mRNAs, miRNAs are also both direct and indirect FOXP3 targets in Treg, and a subset of these miRNAs is also differentially expressed, suggesting a key role for miRNAs in Treg function (76). Selective inactivation of miRNA processing by deletion of the Drosher gene in Treg induced a lethal inflammatory disease in mice, due to a significant reduction in Treg numbers (52), suggesting that miRNAs are required for Treg formation. Furthermore, using Dicer-knockout mice, Liston et al. showed that Treg-suppressive function is absolutely dependent on miRNA biogenesis. These mice showed significantly reduced suppressive activity (54). Further validation of the importance of miRNAs in shaping Treg function was provided by Zhou et al., who demonstrated that depletion of mature miRNAs led to uncontrolled autoimmunity and skewing of iTreg to a Th1/Th2-like effector phenotype. Interestingly, no effect was seen in tTreg (59). This established the possibility that miRNAs may confer a rheostat-like function in T-cell lineage differentiation and plasticity. The potential for the cooperation of miRNAs and FOXP3 enabling tight control of Treg phenotype and function is mechanistically plausible. We and several other groups have demonstrated that miRNAs, such as miR-155 and FOXP3, cooperate to coordinately repress other key genes in Treg and other cell types (77), including SATB1 (34) (Figure 3A), and have identified a number of other candidate miRNAs involved in reinforcing the Treg genotype. We observe that a common miRNA/FOXP3-mediated molecular switch is able to regulate several key genes, and this forms a negative feedforward component shaping part of the FOXP3 GRN.

There is also evidence for positive feedforward regulatory mechanisms in Treg, whereby FOXP3 induces genes while also repressing miRNAs that can target that gene (Figure 3B). A positively regulated miRNA signature for human nTreg feedforward loops includes miR-21, miR-155, miR-125a, miR-146a, miR-181c, and miR-374. There are miRNAs that can target FOXP3 itself, including miR-15a/16 (78), miR-24, and miR-210 (79); however, less is known about the miRNAs which form part of a negative regulatory loop. An miR-31 target sequence in the 3′-untranslated region (UTR) of FOXP3 suggests that miR-31 may be able to negatively regulate FOXP3, and this was validated by overexpression in cord blood nTreg, resulting in a significant reduction in FOXP3. In contrast, antagonism of miR-31 leads to increased expression of FOXP3, suggesting that miR-31 directly regulates FOXP3 (56). miRNA-31 was not identified in the mouse miRNA Treg signature (52), but Zhang et al. identified a potential FOXP3-binding site within the promoter region of the gene encoding murine miR-31 (80), suggesting that FOXP3 may directly target miR-31. Semiquantitative RT-PCR of miR-31 was ~90-fold higher in Tconv than in Treg, and mouse FOXP3 ChIP demonstrated occupancy at the miR-31 promoter (81). Taken together, these experiments suggest that FOXP3 can bind to and downregulate expression of miR-31 in Treg, and by performing an alignment with the human miR-31 host gene, there is also a comparable FOXP3 consensus binding site in human miR-31. Given this potential regulatory rheostat relationship between FOXP3 and miR-31, it is interesting that miR-31 is dysregulated in several autoimmune diseases such as inflammatory bowel disease (IBD) or Crohn's disease (82, 83) and Kawasaki disease (84). Hence, identifying the molecular mechanisms by which FOXP3 and miR-31 regulate each other and identifying the other downstream target genes in this regulatory network could assist in the development of novel treatments for autoimmune diseases.

### Lineage Fidelity and miRNAs

Sentinel transcription factors in each lineage and the miRNAs controlled by them shape CD4+ T-cell phenotypes. An example of the involvement of miRNAs in this is the targeting of mTOR by miR-99a and miR-150 (85), which skews metabolic processes and influences levels of FOXP3 and RORgamma (86). This may enable switching between functional phenotypes by driving one transcription factor to decline and another to dominate, which is a potential molecular mechanism for plasticity. In addition, it is interesting to speculate that the transient expression of FOXP3 in activated Tconv then induces miRNAs, which together transiently repress effector function gene networks, and this enables the effector cells to return to a resting state.

### A Role for Long Non-coding RNAs in Shaping Treg Function

In addition to short non-coding RNAs, high-resolution functional annotation of the human and mouse genomes has revealed the prevalence and importance of long non-coding RNAs (lncRNAs), which also act as subtle regulators of gene expression. lncRNAs (defined as non-coding transcripts >200 bp) are not translated but can regulate gene expression as a result of interaction with mRNA and chromatin. This is achieved by either stabilizing DNA looping or by integrating into the RNA-binding protein complex to regulate transcription (87). The lncRNAs have been implicated in differentiation of T-cell subsets (88) and in immune function (89, 90). Alterations in lncRNAs function have been identified in autoimmune and chronic inflammation samples (91). In keeping with miRNA feedforward and feedback loops, the importance of lncRNA in Treg has been elegantly demonstrated for an lncRNA (FlicR) in mouse and human Treg. FlicR stabilizes the expression of FOXP3 (92) via interactions with conserved non-coding elements that control FOXP3 expression, and loss of FlicR results in reduced expression of FOXP3. Although it is not clear what regulates transcription of FlicR itself, controlling FlicR could establish the transcriptional reprogramming of Treg. If its expression was susceptible to external cues such as IL2 signaling, this would support a model that plasticity can be induced by relatively small changes in signaling that result in altered transcriptional networks. It is also interesting that a role for lncRNA in stabilizing DNA looping has been proposed. Consistent with this, lncRNAs are often encoded within enhancer regions, which shape the expression of multiple genes. Since a significant proportion of autoimmune genetic risk is also found in enhancers and the enhancers loop to form the regulatory hubs for key immune function genes, this suggests that there could be a complex network effect on multiple targets from genetic risk at a single lnc/enhancer module (93).

### Translational Regulation and Treg Phenotype

In addition to transcription and posttranscriptional regulation of the Treg phenotype, there is a layer of control of the Treg or T effector phenotype at the translational level. This is influenced by activation and TCR crosslinking and is mediated by ribosome occupancy levels on mRNA and the expression of translational machinery, which can differentially impact protein levels in the cell. One member of the transcriptional initiation complex is elongation initiation factor 4E (eIF4E), and its expression inversely correlates with FOXP3 expression (94). A cluster of effector cytokine genes is positively regulated by eIF4E and is expressed by activated Tconv but is repressed in Treg. It is interesting to note that IL2 signaling can induce eIF4E, but in Treg, the expression of FOXP3 can repress this. Given that Treg are dependent on exogenous IL2 for survival and proliferation signals, but Teff can express IL2, there are likely additional layers of regulation that prevent IL2-induced gain of effector function in Treg. This may include the regulation of some of the downstream signaling by mTORC. Hence, a disease-linked alteration of eIF4E levels in Treg is likely to reduce FOXP3 expression and unleash effector cytokine expression, driving the switching of phenotypes (plasticity or immune defect) (95).

### Treg Gene Expression Is Set by Enhancer–Promoter Interactions

Genes comprise as little as 1.5% of the human genome, encoding ~21,000 proteins, leaving 98.5% of the genome that is non-coding, and this is responsible for orchestrating the cell-specific expression of genes required for formation and function of every cell in the body. It is now clear that interactions between coding and non-coding elements are essential for normal gene regulation and maintenance of stable phenotypes. To achieve this in the contact of the relatively compact nucleus, chromatin structure has a major influence on gene expression by controlling transcription factor access to binding sites in enhancers and promoters (96). This is shaped by patterns of chromatin modification at the base pair level, such as DNA methylation, or the macromolecular level, such as histone modification and nucleosome remodeling, and these correlate with transcription factor binding, enhancer activity, and initiation or repression of transcription (97–99). There are additional, highly active enhancer clusters named super-enhancers (100, 101), and they appear to regulate key genes involved in T-cell function. It is now necessary to consider how enhancers interact with their target genes, particularly as they can be significant distances apart on linear DNA. DNA looping promotes gene network formation, and a single enhancer can interact with more than one promoter, and a single promoter may be contacted by more than one enhancer. As this 3D chromatin organization is being unraveled, it appears that many of these interactions occur in a tissue-specific manner and are the major determinants of cell-type-specific responses (102–105).

### Transcriptional Control by DNA Looping

DNA looping brings specific genes and regulatory elements together into transcriptionally active hubs (106), and these hubs may be different in Tconv or Treg. However, since DNA looping cannot currently easily be predicted using bioinformatics approaches, proximity-based annotation of FOXP3 targets based on linear DNA organization under-ascribes FOXP3-binding sites in chromatin to transcriptional targets. This has provided a partial explanation for the apparently low intersection of transcription factor ChIP peaks (including FOXP3) with differential expression of target genes in the same cells, as those interactions have traditionally been annotated using a linear nearest-neighbor approach.

New techniques have been developed to solve the problem that bioinformatics alone is not readily able to predict long-range DNA looping. These techniques are collectively known as chromosome conformation capture (3C) assays, and these are essential to study the role of DNA looping in transcriptional regulation. 3C is able to efficiently crosslink looped DNA for barcoding, and by using sequencing and mapping of non-contiguous sequences, it is possible to directly identify genomic loci that are in interaction partnerships over short and long distances (107). Variants of 3C such as 4Cseq, ChIA-PET (108), and 5C (103) can identify individual promoter interactions (3C), the network of interactions with one bait locus (4C), the interactions between a transcription factor bond to DNA ant its contacts (ChIA-PET), and single-cell conformation capture (5C). These methods have been extended to examine interactions in an unbiased genome-wide manner, HiC (102). HiC enables mapping of whole-genome chromatin interactions, although there is currently little confidence in the statistical power for predicting interchromosomal interactions. As HiC aims to annotate any/all contact between any two loci on a genome-wide level, it requires deep sequencing to generate comprehensive coverage and to generate interaction maps at the resolution required to map both near and far contacts with accuracy. Given that a significant number of interactions involve a promoter, as that is essential for regulating gene expression, modifications of HiC to reduce sequencing depth and to focus the sequencing to regions of functional interest have been derived. This is achieved by adding oligonucleotide capture technology to enrich regions of interest (such as promoters) in the HiC library prior to high-throughput sequencing (109–111). An advantage is that the HiC library can be re-probed with different oligo libraries, e.g., enhancers, promoters, or ChIP sites, and this enables validation of promoter capture by reverse capture from the same cell source (102). ChIP combined with HiC enables generation of specific protein-centric interactome maps (112), known as HiChIP (113). When H3K27ac HiChIP was performed in naïve T cells, Th17, and Treg, it shed new light on the lineage-specific interactome by annotating lineage-specific accessible chromatin interacting with regulatory elements (114). Importantly, 3C-based assays have been used to successfully identify targets of disease-associated variation in many cell types (103, 108, 110, 115) including human CD4 and CD8 populations (116). By adding DNA looping, enhancer annotation, and FOXP3 binding data to genetic risk data, it is possible to filter genetic risk to Treg-specific functional regions using bioinformatics. However, functional validation of these regions still needs to be performed on human Treg.

### Chromatin Accessibility Controls Gene Regulation in a Cell- and Activation-Specific Manner

Annotation of active, open, or closed chromatin has facilitated mapping of cell-type-specific gene regulation [e.g., epigenomics roadmap (97, 117) and FANTOM (98, 118–120)]. These consortia have provided additional evidence to map autoimmune disease in the context of the activation state, connectivity, and accessibility of the genome. ATACseq (Assay for Transposase Accessible Chromatin with high-throughput sequencing) probes chromatin accessibility, TF occupancy, and nucleosome positioning with low starting material (121, 122). As compared to other genome-wide chromatin probing methods, ATACseq is relatively simple and rapid. It is also highly sensitive. The requirement of low-input material makes ATACseq amenable to use on rare population and small clinical samples such as biobanked material (123). At a high-sequencing depth ATACseq can also be used to identify TF binding sites at single-base-pair resolution. TF-occupied sites prevent Tn5 cleavage and adaptor insertion, thus leading to protected regions (footprints) in the sequencing reads (121). This technology is enabling the base pair level mapping of the potential impact of genetic risk on gene regulation, as a SNP that alters a TF binding site will alter the profile of the ATACseq signal at that region, compared with the non-variant base at the same locus. In addition, chromatin accessibility profiling in human Treg enables identification of cell-specific accessible regions that contain features such as FOXP3-binding sites and genetic risk, and these can be distinguished from regions nearby that may also contain genetic risk but are not active in the cell type of interest.

### Environmental Signals and Transcriptional Programming of T Cells

#### Sensing External Stimuli at Sites of Inflammation

The process of recruitment of effector cells to pathogens in the host tissues is driven by local tissue cues drawing the cells to the site as well as pathogen recognition signals, and this is in part mediated by the activation of the pro-inflammatory milieu at these sites. This suggests that local signals may contribute to the shaping of the phenotype of the cells once they home to the challenge site (73, 124). It is also common that the tissue site has altered metabolic status, such as hypoxia and altered redox states. It is now evident that T cells are also highly responsive to these metabolic cues, and these are sensed by common surface receptors and biochemical pathways. The potential for altered regulation of the immune response at these sites exists because the Treg have to home to the same locations to regulate the effector response after pathogen has been cleared, and they are also exposed to the same metabolic environment. For robust regulation by Treg in this context, the FOXP3 GRN has to resist environmental fluctuations (125). However, it is also possible that tissue-specific cues can transiently reduce Treg function and limit their suppressive potency, in order to enable pathogen clearance. Taking into account that strong T-cell activation can also induce transient FOXP3 in effector cells, this may be the mechanism by which the effector cells themselves return to a resting state, but this has yet to be proven.

Transcriptomics has revealed micronutrient transporters and receptors on human T cells, rendering them responsive to a wide range of metabolic molecules and signals. These include sugars, amino acids, environmental toxins (126), energy molecules, vitamin metabolites, and food metabolites, many of which are processed by the microbiome. Functional validation and characterization at the molecular levels suggest that these pathways are particularly relevant for induced Treg generation in the gut. These mediators play a key role in differentiation of naïve T cells, but it remains to be definitively proven that they can drive fate change in committed T-cell subsets. Furthermore, T cells can sense oxygen tension and oxidation state, and these also exert a phenotype-altering potential as they in turn regulate gene networks influencing biochemical responses including glycolysis. As described below, the balance of oxidative phosphorylation vs. glycolysis is linked to transcription of FOXP3 and regulatory phenotypes (127, 128). This in part enables Treg to function in environments that are under oxidative stress (129) and also suggests that metabolic status could skew immune function.

A number of specific metabolites which can alter transcriptional programming and T-cell differentiation have also been identified. For example, FOXP3 can be induced directly in response to short-chain fatty acids processed from complex carbohydrates, e.g., starch, in the colon by specific microbiome constituents. Hence, butyrate processed by commensals in the colon is able to promote a tolerogenic bias (130, 131). Other metabolites that have been well-characterized include the vitamin A metabolite retinoic acid (ATRA) either alone or in combination with other factors. ATRA can induce either Treg or Th17 phenotypes by upregulating FOXP3 expression or RORgamma expression, respectively (132). This tolerance/inflammation axis can also be influenced by sensing toxins and pollutants via the aryl hydrocarbon receptor (AHR) (133, 134). Mechanistic insight comes from functional mapping of the genes and pathways of the sensors of metabolic stimuli, including mTor and HIF (135, 136). The impact of high salt on Treg function and plasticity has been postulated, and a key mediator of responsiveness to high levels of sodium is the serum/glucocorticoid-regulated kinase (SGK-1) (137). The molecular mechanisms of SGK-1-mediated regulation of Treg suppressor function have been validated in knockout models, and this demonstrated that SGK-1 is induced by IL23/IL23R signaling, and elevated levels of SGK-1 result in reduced suppressor function, which is linked to reduced FOXP3 expression, and that is caused by reduced binding of FOXO1 to the CNS1 element in the FOXP3 promoter, described above. The induction of SGK-1 therefore induces a transcriptional bias to Th17 cells. Knockout of SGK-1 results in enhanced Treg function and reduced pathology in EAE models, confirming a functional link to immune tolerance balance (138). All of these pathways and inducer molecules are implicated in altered Treg function or numbers in disease and are potential targets for interventions to restore balance.

### Energy Pathways and T-Cell Function

Long-lived quiescent T cells primarily utilize oxidative phosphorylation pathways as their energy source and upon activation switch to glycolysis (139–141). Glucose transporters GLUT1 and also GLUT3 and GLUT4 are rapidly induced and traffic to the cell membrane to promote glycolytic metabolism and cell growth (142). However, not all differentiating T cells have the same energetic requirements and in fact have quite distinct metabolic programs (143). Although T cells differentiating into Th1, Th2, and Th17 cells reprogram their metabolic pathways by turning on glycolysis, Michalek et al. first showed that differentiating Treg exhibit a unique metabolic profile relative to other CD4+ effector subsets. In this study, the Treg did not induce glucose transporters and upregulate glucose uptake and glycolysis; however, the Treg were not quiescent, and instead, their mitochondrial membrane potential increased, and lipid oxidation likewise increased. Insight into the Treg response to glycolysis, including transcriptional programming of splice variants of FOXP3 itself, has provided insight into loss of Treg function in autoimmunity and demonstrates that control of FOXP3 expression is important for stable suppressive function (144, 145). Recently, Weinberg et al. (146) have added to this, demonstrating that mitochondrial complex 3 is essential for Treg suppressive function. In mitochondrial complex 3-deficient mice, FOXP3 expression itself is not altered. However, immune regulatory gene expression and suppressive function were ablated. Fatty acid pathways have been linked to mitochondrial integrity, and this in turn impacts suppressive capability, and, for example, inhibition of the fatty acid binding protein 5 (FABP5) enhances suppressive function mediated by IFN1 signals and IL10 induction (147).

### mTOR Signaling in CD4 Tconv and Treg

The kinase mTOR is activated upon CD4+ T-cell activation and has a pivotal role in the management of crucial cell functions, sensing a range of environmental cues such as cytokines, growth factors, and nutrients to regulate metabolism, protein synthesis, proliferation, and survival (148–150). mTOR signaling takes place via two complexes mTORC1 and mTORC2, whose regulation and activities are somewhat distinct from each other. An essential component of the mTORC1 complex is the scaffolding protein, regulatory associated protein of mTOR (RAPTOR). Activation of mTORC1 through PI3K-Akt signaling pathways has a central role in regulating T-cell growth and proliferation (148, 150) and has been shown to be required for correct differentiation of Th1 and Th17 cells, while mTORC2 and its essential subunit protein rapamycin-insensitive companion of mTOR (Rictor) are vital for Th2 differentiation (148–150). Activation of mTOR suppresses Treg development (151) while mTOR-deficient cells (148, 150) or cells with a blockade of glycolysis (152) differentiate into Treg. However, mTORC1 signaling is a pivotal positive determinant of Treg function under steady state and immune stimulation (153). mTORC1 activity coordinates the increase in CTLA4 and ICOS expression in Treg to upregulate their suppressive activity, orchestrating the lipogenic program. Raptor-deleted mice develop severe autoimmune disease, which demonstrates that this is absolutely required. There is hence a fundamental role for Raptor/mTORC1 in cholesterol and lipid biosynthesis, highlighting the mevalonate pathway as important for coordinating Treg proliferation and induction of effector molecules CTLA4 and ICOS. In addition, there is a role for liver kinase B1 (LKB1) in coordinating intracellular cholesterol biosynthesis via the mevalonate pathway in Treg cells, further demonstrating this pathway as crucial in the inhibition of inflammatory cytokine production and promotion of the suppressive activity of Treg (154). Thus, fine rheostat control of lipid metabolism is crucial for the optimal programming of suppressive activity, immune homeostasis, and immune tolerance in Treg cells (143). With the use of a knockout mouse model, the importance of these pathways in suppressor function was recently highlighted, demonstrating that the absence of FOXP3 antagonism of mTOR promoted suppressor function (155).

### Plasticity Driven by the Metabolic Program of Treg and CD4+ T Cells

**Figure 4:**
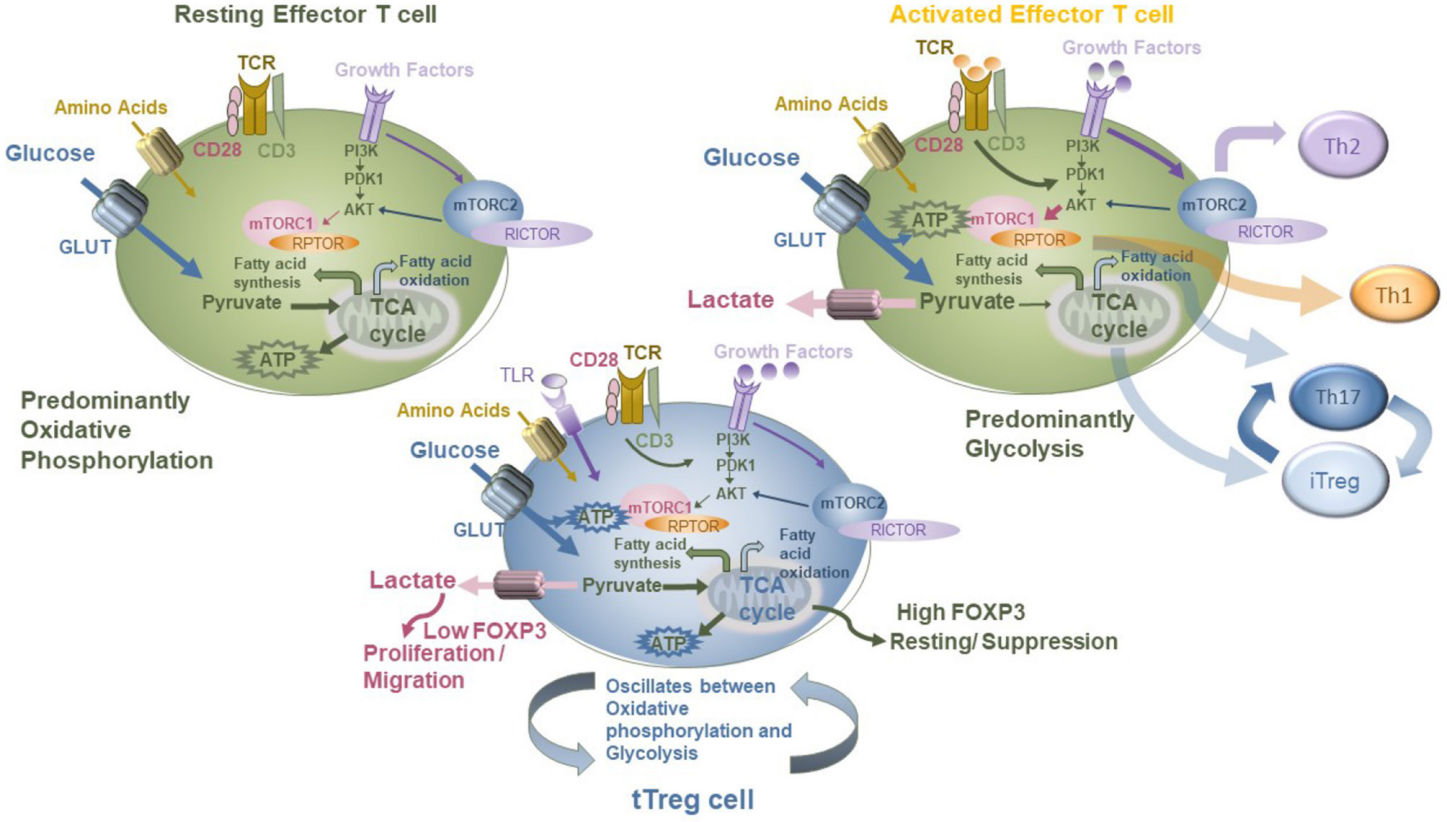
Modeling the metabolic modifiers of T-cell function in Tconv and Treg, showing differential impacts of glycolysis and oxidative phosphorylation on each and the role of the mTOR pathway in mediating this.

Treg are clearly more pleiotropic than previously envisaged. Their metabolism may oscillate between mTOR-dependent and mTOR-independent pathways in response to environmental cues (144) depending on whether they are receiving signals to differentiate, proliferate, or carry out suppressive functions. The control of energy metabolism through the leptin–mTOR pathway in Treg sets their state of responsiveness, and this may be necessary for entry into the G1/S phase of cell cycle and proliferation. Recently, Pryadharshini et al. found that Treg metabolism is reprogrammed depending on whether the Treg are thymic derived (tTreg) or induced (iTreg). Inducible Treg are dependent on mitochondrial oxidative phosphorylation, whereby FOXP3 suppresses glycolysis. In contrast, tTreg engaged in glycolysis more comparable to that of effector T cells. Thus, the different Treg subsets utilized mTOR-dependent and mTOR-independent signaling pathways (156). Treg cells express several Toll-like receptors (TLRs), and these are critical for correct Treg homeostasis and function. Gerriets et al. (157) showed that as Treg proliferate, Glut1 levels increase, mTOR is activated, TLR1 and TLR2 are ligated, but at the same time FOXP3 is downregulated and suppressive activity reduced. Transgenic expression of Glut1 reduced Treg suppressive capacity and downregulated FOXP3. Conversely, FOXP3 diminished PI3K-Akt-mTOR signaling and Glut1 expression. Thus, TLR signals and FOXP3 counter-regulate Treg cell metabolism to balance proliferation and suppressive function. This is one mechanism by which a Treg homing to a site of inflammation is temporarily shut down if the local pathogen load (bacterial lipopolysaccharide level) is high, but once the TLRs are no longer engaged, the Treg population regains suppressor function. Shifting the balance of metabolic control can direct T-cell differentiation to specific lineages which can be part of normal immune control but in dysregulation can also lead to immune pathogenesis. FOXP3-deficient Treg acquire effector-like characteristics and lose suppressive function, dysregulating mTORC2 pathways and upregulating glycolysis. Deletion of an mTORC2-associated protein, Rictor, can re-establish partial Treg phenotype by restoring suppressive function to the impaired Treg (155). This restoration of Treg function opens up the possibility of reprogramming the metabolism of deficient Treg (such as in IPEX disease where mutations to FOXP3 are common) by targeting particular metabolic pathways (summarized in Figure 4).

### Treg in the Peripheral Tissues

Tissue-resident Treg are found in almost all tissues including visceral adipose tissue (158) and skin and have unique transcriptional programs enabling them to home to and reside in these locations (159). Tissue Treg are frequently associated with damage repair and are activated or expanded in response to damage. This is mediated by alarmin signaling and induction of tissue-specific tissue repair signals, e.g., amphiregulin. The IL33–ST2 signaling partnership is key to tissue Treg function, where local expression of IL33 or IL1 has opposing effects on Treg polarization and hence function (160–162), giving fine-tuning of Treg function in the tissue in response to injury. There is hence a growing role for tissue-resident Treg, such as VAT Treg (163); however, some of the tissue repair function is independent of suppressor function (7). This is worthy of fine analysis as localized inflammation is also suppressed by tissue Treg, but it is not yet clear if this is a heterogeneous mixture of subsets in the tissue or a dual function of a single subset (164). ST2+ Treg are implicated in tumor tolerance, and this may be an underappreciated consequence of their role in tissue repair (165). It is of some clinical relevance that tissue repair by Treg in zebrafish is able to reverse organ damage (166), but in the context of autoimmune damage in mice or humans, this capability is lost.

### Treg and Immune Disease

It is possible that under specific circumstances, the balance of Treg to effector lineages may be altered or that the Treg reprogram and switch fates. Given the complexity and connectedness of the Treg GRN, there are many points that alter Treg or Tconv function, many of which could be affected by genetic or environmental risk factors. It is relatively rare to find mutations in FOXP3 itself (IPEX), suggesting that Treg-specific defects in autoimmune disease are likely to result from reduced FOXP3 function or alterations in expression of downstream targets, but not as a result of sequence changes in the FOXP3 gene body. This is further complicated by the observation that there are also genes involved in Treg function that are FOXP3 independent. It is interesting to note that when standard Treg flow cytometry data are analyzed using tSNE algorithms, three distinct FOXP3 populations can be resolved, and this is more complex than can be observed by 2D FACS analysis. Decreased Treg numbers or impaired Treg function in adult mice can cause autoimmune diseases (1), and the mechanisms and drivers of this have been revealed using numerous gene-targeting and fate-mapping models that also develop disease when the Treg compartment is perturbed. The therapeutic potential of inducing or restoring tolerance has also been demonstrated using adoptive transfer of Treg, which ameliorates many symptoms in non-obese diabetic (NOD) and IBD mouse models (1), as well as mouse models of pregnancy disorders, which mimic autoimmune disease in many regards (167). In humans, this is mirrored in IPEX patients who lack FOXP3 and Treg (28), and the early development of autoimmune disease in IPEX confirms that Treg are also essential in humans (168). Taken together, these data suggest that a threshold of Treg function is required throughout life to restrain autoreactive T cells and/or inflammatory responses, and loss control of this process licenses autoimmune disease onset. Nonetheless, there are conflicting reports in the literature about reduced Treg numbers in clinical autoimmune cohorts. This may be caused in part because of evolving biomarker combinations used and methods for enumerating Treg and impacted by the need to consider the amount of FOXP3 as well as the absolute presence or absence of FOXP3+ cells in flow cytometric data. This was also confounded by the observation that activation of T cells induces FOXP3 transiently in cells that are not Treg. We and others have demonstrated that loss of FOXP3 expression levels, rather than reduced absolute cell number, is observable in autoimmune cohort samples and may be a precipitating factor for reduced immune tolerance in these cohorts (24).

In Treg, a consequence of loss of FOXP3 expression as a result of transcriptional or translational defects could be reduced Treg function. This might be triggered by exhaustion or chronic overstimulation, such as could happen during a potent immune response, and this has led to the concept of ex-Treg. Fate-mapping studies elegantly demonstrate that, in mice at least, Treg can lose expression of FOXP3, but they are demonstrably of thymic Treg origin, based on genetic marking. These studies implicate ex-Treg in susceptibility to multiple sclerosis (169) and rheumatoid arthritis (170) and suggest that the high levels of IL6 at sites of tissue damage and inflammation can induce this loss of FOXP3 expression *in vivo*. As IL2 signaling is repressed by SOCS1 (171) and SOCS1 is induced by IL6 signals, this may be a contributing factor for reduced FOXP3 expression in pro-inflammatory scenarios. We and others have reported elevated IL6 and IL1, among other pro-inflammatory cytokines, in the local tissues in autoimmune disease samples including IBD (172, 173). Hence, a second axis driving plasticity may be the impact of local pro-inflammatory cytokines including IL6. Mechanistic insight into this has emerged recently with the identification of a huTreg subset that expresses gp130, the common gamma chain of the IL6 receptor, and the finding that these gp130^+^ Treg are less suppressive and express lower levels of FOXP3 (174). The loss of tissue-resident Treg function is also associated with pathology and observed in almost any non-lymphoid tissue, and these include the lungs (175, 176), liver (177), and skin (178), and this is the result of either Treg intrinsic defects or altered IL33 signaling, such as that found in allergen-sensitive airways (176).

### Environmental and Genetic Risks Combine to Alter Immune Function

The observation that many autoimmune diseases are the result of the intersection of genetic risk and external environmental triggers comes from the fact that there is incomplete penetrance in all autoimmune diseases, and this is in spite of the presence of genetic risk. Type 1 diabetes (T1D) is an example of a disease which arises as a result of complex interactions between genetic and environmental factors conspiring to drive the pathology, resulting in disease progression. In a meta-analysis of six independent genome-wide association studies (GWAS), each aiming to identify single-nucleotide polymorphisms (SNPs) that track with T1D, ~45 loci were enriched (179, 180). We hypothesize that as the disease linked to a failure of self-tolerance, Treg cells would in some way be impacted by this genetic risk, so we intersected the genetic risk loci with our FOXP3 ChIP data (57). This revealed that 34 (>70%) contain a FOXP3-binding site, which is significantly above the genome-wide distribution of FOXP3-binding sites (~15%). Hence, the enrichment of T1D genetic risk in regions that are potentially directly controlled by FOXP3, and therefore actively regulated in Treg, suggests potential for a Treg-specific defect directly as a result of polymorphism in regulatory elements controlling these genes.

As described above, genome-wide mapping of epigenetic variation in Treg and Tconv suggests the potential for cell-type-specific transcriptional activity, but this is not restricted to T1D (181, 182). From numerous GWAS datasets, there is very strong probability data linking genetic variation (SNP) to a wide variety of immunological disease cohorts. It is clear that the majority of this variation is not in the coding regions of the genome but in specific non-coding regions enriched for regulatory elements such as promoters and enhancers (101). When this is nuanced with datasets that have functional annotation, the majority of this non-coding genetic variation overlaps and thus likely influences transcription factor binding sequences and lncRNAs (183–185). Therefore, the functional impact of genetic risk has to be studied in the cell type driving diseases. It is not possible to accurately define this in cell lines from other tissues. With regard to GWAS datasets derived from autoimmune cohorts, the significant enrichment in T-cell-specific promoters and enhancers (100, 185) suggests that perturbation of gene regulation in multiple pathways in the immune system can result in the same phenotype from unrelated genotypes.

As this is a bioinformatics-based intersection, the functional link between them is currently unknown. The same genetic risk is carried in all CD4 effector T-cell populations, and although many cell types contribute to immune homeostasis, Treg/Tconv defects play a major role in the pathology of human autoimmune disease. Thus, it is plausible that both effector and Treg are impacted by genetic variation. Susceptibility to disease is therefore linked to Treg plasticity, altered Treg development, or altered Treg function, and this can be therapeutically targeted once the pathways are identified. For example, TNF antagonism has been demonstrated to be effective on Treg in rheumatoid arthritis (186).

## Conclusion and Future Directions

**Figure 5:**
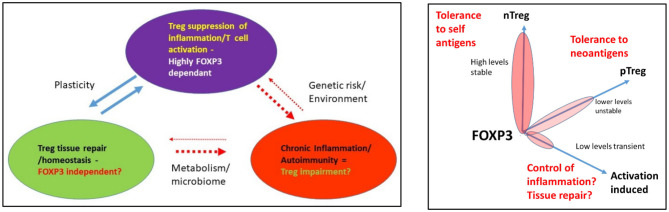
**(A)** Functional roles of Treg and the impact of environment and genetic risk. **(B)** Modeling a functional link between FOXP3 levels and biological process.

There are complex dynamic regulatory processes controlling Treg generation and stability that involve the interplay between transcription factors, miRNAs, and lncRNAs to shape Treg-specific regulation of gene expression. These occur between genes and enhancers over long and short distances and are only active in regions of open chromatin. Given that master transcription factors shape almost every lineage in the lymphoid compartment, it is plausible that these interact to set the expression levels of the transcription factors which themselves define function. Importantly, as T-cell function is dynamic and responsive to external cues, the enhancers and super-enhancers establish the level and kinetics of gene expression both in the steady state and in response to these cues. This gives three layers of reinforcement of Treg phenotype in the normal context. Each may be a point of disruption, either in disease, which alters the numbers or function of the Treg pool, or in response to appropriate tissue and inflammatory cues, which alters cell fate transiently. If this is a programmed change rather than induced by disease risk, it may be described as plasticity. Plasticity is hard to demonstrate in humans, as it is difficult to accurately model human T-cell fate in real time (Figure 5A).

Given that FOXP3 establishes and maintains a strong regulatory phenotype in healthy individuals that is resistant to reprogramming, there is potential for this to be disrupted under specific circumstances, e.g., in carriers of disease-associated genetic risk. The result could be the formation of a cell with an effector-like function that expresses less or no FOXP3. It is also plausible that the organ-specific damage in autoimmune diseases such as T1D may be a result of the dual impact of the loss of suppressor function resulting in an inappropriate anti-self-immune response, as well as a failure of the tissue repair capacity of the Treg, resulting in loss of beta cells (Figure 5B). However, using the newest genomics and high-resolution cell phenotyping, the question of identifying mechanisms underpinning loss of function in human Treg will likely soon be answered. This will necessarily require functional validation in human T-cell subsets. Methods including gene editing are a powerful tool for pathway functional validation, including engineering of genetic risk into healthy T cells to assess its impact. It can also be used to target miRNAs and lncRNAs to assess their role in fine-tuning alterations in genotypes required to alter phenotypes and to perhaps confirm these are the rheostat of fate. This will also reveal if dysregulation of miRNAs is a tipping point for altered phenotypes. In time, these approaches will provide diagnostic information and new points for therapeutic intervention to reverse the impact of genetic risk on gene expression in Treg and Tconv in many diseases, including autoimmunity.

## Author Contributions

CB and TS contributed to writing, editing, and performed some of the work referred to in this review. YW, SW, NL, HW, KB, VB, and CH contributed to editing and figures generated for this review. SP and JB contributed bioinformatics analysis, editing, and experimental design input to this review. SB, MB, AF, and SR conceived, contributed to writing, and edited the work. All authors contributed to the article and approved the submitted version.

## Conflict of Interest

The authors declare that the research was conducted in the absence of any commercial or financial relationships that could be construed as a potential conflict of interest.
